# Effect of cold stress on infanticide by female Swiss albino mice *Mus musculus*: a pilot study

**DOI:** 10.1186/s40781-018-0168-6

**Published:** 2018-04-23

**Authors:** Tabassum Zafar, Ab Qayoom Naik, Vinoy K. Shrivastava

**Affiliations:** Laboratory of Endocrinology, Department of Biosciences, Barkatullah University, Bhopal, M.P 462026 India

**Keywords:** Cannibalism, Mice stock maintenance, Infanticide, Pups mortality, Cold stress

## Abstract

**Background:**

Mice are widely accepted research models of great clinical significance. Maintenance of laboratory mice breed is an essential aspect for performing research activities in various fields of science. Infanticide is one of the prominent causes of litter loss during maintenance of laboratory mice stock. The present study is an effort to monitor the effect of change in ambient temperature of female mice below the normal range on cannibalism and infanticide during early postparturition phase. Adult female Swiss albino mice have been divided into two groups of control and treatment. On the day of litter group one was maintained under controlled temperature conditions (minimum 20 °C to maximum 23 °C) throughout, while female mice belong to group two have been exposed to variation of room temperature (maximum 15 °C to minimum 10 °C for two nights and one day) until 36 h postparturition.

**Results:**

The effects of temperature changes were observed on the infanticide behaviour of dams along with the survival of pups in early postparturition phase till 36 h after delivery. The significant statistical difference (*P* < 0.05) was reported in infanticide behaviour of dams when control and treatment group was compared. It is observed that decrement in surrounding temperature promotes decrement in the ambient body temperature of dams during early postparturition. It is proposed that alteration of hypothalamic homeostasis due to temperature change induces cannibalism and infanticide behaviour. Lack of thermoregulation during early postparturition creates the sense of insecurity, in-satiety, anxiety and stress.

**Conclusions:**

Authors strongly recommend the maintenance of body and surrounding temperature to prevent infanticidal behaviour and cannibalism within Swiss albino mice population. Further investigations are advisable to authenticate the active behavioural and biochemical pathway behind the phenomena.

## Background

Cannibalism is the phenomena in which one individual of a species consumes whole or parts of the individuals of the same species. It is widely seen in rodents, insects, and lower vertebrates. Cannibalism in the form of infant consumption is termed as infanticide. Infanticide refers to the act of killing infants by their own parents [1]. Swiss albino mice *Mus musculus* is a widely used mouse strain, which is used for many research investigations due to its small size, short life span, and easy maintenance. Maintenance of mice stock is an essential need for research facilities due to their widespread use in clinical studies related to biomedical research. Importance of mice as a research model is not only limited to toxicity assessment, drug delivery, stem cell research, cancer biology, endocrinology, and infertility assessment but also extended beyond the imagination [2, 3].

Successful mice breeding are crucial aspect of mice stock maintenance and many other experiments such as mating assay and fertility assessment. Any direct or indirect modifier can lead ambiguities in experimental findings of researchers. Loss of pups or entire litter loss by infanticide behaviour of dams is relatively a common problem, which is poorly understood. However, there are various hypotheses available on the subject but still the early postnatal mortality is a subject of interest for further investigations. Any specific change, which is even non-noticeable for human could affect the pregnant mice effectively. It is assumed that any kind of behavioural changes due to stress or malnutrition can affect the dams and pups negatively [4]. Extend of postpartum stress response of mice depends on the duration, consistency and nature of stressors. Hence, it is very important to manage the dam and pups under controlled conditions with consistent monitoring of postnatal death rate [5, 6]. Present study is an approach to access the possible effect of cold stress exposure on infanticide behaviour of dams during early postparturition phase in inbred mice population.

## Methods

Animal care and handlings were performed according to the guidelines issued by CPCSEA (Committee for the purpose of control and supervision of experiments on animals), New Delhi, India. The present study is a part of study approved by institutional ethical committee of Barkatullah University, Bhopal and CPCSEA, (Committee for the purpose of control and supervision of experiments on animals) New Delhi, India.

Adult female Swiss albino mice were purchased from Jawaharlal Nehru Cancer Hospital and Research Center, Bhopal, India and housed in Polypropylene cages containing paddy husk (procured locally) as bedding. After the successful completion of quarantine period twelve adult female albino mice of similar estrus stage were housed with four male albino mice inside the animal facility of Department of Biosciences, Barkatullah University, Bhopal, India for the assessment of reproductive potential and reproductive fecundity. After the presence of copulation plug pregnant females were separated from males. The mice were maintained under controlled conditions of temperature and light along with standard mice feed and water ad libitum throughout. During the time of mating the average room temperature of animal house was maintained 22-23 °C. On the day of litter control group (*n* **=** 6) was maintained under controlled temperature conditions (minimum 20 °C to maximum 23 °C for two nights and one day), while female mice belong to treatment group (n **=** 6) have been exposed to variation of room temperature (maximum 15 °C to minimum 10 °C for two nights and one day) till 36 h postparturition. After the successful completion of 21 ± 1 gestation period, pregnant female mice delivered the pups. During that time the effects of temperature changes were observed on the infanticide behaviour of dams along with the monitoring of pups survival upto 36 h postparturition [7].

The collected data were subjected to statistical analysis using Excel–mac operating system software. Mean ± standard deviation and standard error of mean were calculated. Independent student’s ‘t’ test was used for statistical comparison and significance level determination between the control and treatment groups using Excel–mac operating system. *P* < 0.05 is considered as significant.

## Results

During the present study it was observed that the female mice belong to control group, which were maintained under standard ambient temperature postparturition have shown normal maternal behaviour with a high percentage survival rate of infants within first 36 h of the delivery. However, dams belong to treatment group, which were exposed to temperature variation postparturition (maximum 15 °C to minimum 10 °C for two nights and one day) have shown signs and symptoms of stress along with potential infanticide behaviour (Table 1).

**Table 1:** Details of born and survived pups

Pups born in control group (Mean ± SD)	Pups survived in control group 36 h postpartum (Mean ± SD)	Pups born under cold stress treated group (Mean ± SD)	Pups survived under cold stress treated group 36 h postpartum (Mean ± SD)
5.5 ± 1.048	3.66 ± 1.032 ^a^	5.16 ± 1.169	0.5 ± 0.54^a^

^a^denote statistically significant difference between groups (*P* < 0.05)

**Fig. 1:**
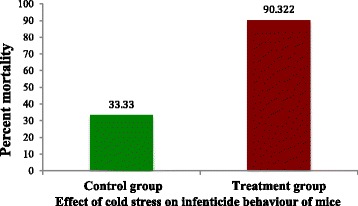
Effect of cold stress on infenticide behaviour of mice

Number of dead pups and total number of pups born in each group were observed carefully for the assessment of percent mortality (Fig. 1). The significant statistical difference (*P* < 0.05) was reported when pups mortality compared between control and temperature altered treatment group. Pups belongs to treatment group have been shown significantly higher mortality in compare to pups of group maintained under controlled temperature (Table 2).

**Table 2:** Statistical details of mortality reported among control and treatment groups

Pups mortality in control group (Mean ± SD)	Pups mortality in cold stress treatment group (Mean ± SD)
1.833 ± 0.752 ^a^	4.66 ± 1.211 ^a^

^a^denote statistically significant difference between groups (*P* < 0.05)

Significantly less pups survived until 36 h postpartum in altered temperature conditions; however, dams belongs to control group, which were maintained under controlled conditions of temperature throughout have raise their litters better with significantly higher pups survival (*P* < 0.05).

## Discussion

Maternal infanticide has been suggested to be among the main causes of perinatal mortality. In spite of various investigations available in literature to address the questions related to cannibalism and infanticide behaviour after parturition, it remains an unsolved puzzle. Existing literature and explanations gave rise many rationale, which depict the effect of many conditions on cannibalism and infanticide in mice. Among other laboratory rodent, rats and mice are more likely supposed to consume their abnormal or defected infants. Loss of nutrients in the diet by sterilization or malnutrition were also reported to be responsible for a high percentage of pre-weaning deaths [8]. Many research investigations indicate that caging and animal house lighting must be considered in relation as opaque cages are better choice for breeding mice similarly dim lights are more likely to prevent infanticidal behaviour. However, mice age is not related to infant consumption but evidences are available in relation of better breeding outcomes in ninteen and twenty four weeks old mice with successful weening of pups [9].

Dams show their maternal care by nursing the infant, keeping them warm, protect them from other immediately following the delivary [10]. An interruption or disturbance of mothering behaviour is likely to lead towards neglect, death and sometimes eating of young in nest [11–14]. These postpartum behaviours are triggered by hormonal changes during late pregnancy and also by the presence of pups after delivery. Many factors such as hormones affect the pre optic neuclei, hypothamus and amygdala. These brain regions have very important and active role in maternal behaviour, nursing and affection [15–17]. Hypothalamus is known to play a vital role in thermoregulation. The nerve pores present in skin transmit the signals to brain and hypothamus to regulate the temperature [18]. During the completion of signaling pathway other functions of hypothalamus such as hormonal association of maternal behaviour also modulates [19].

**Fig. 2:**
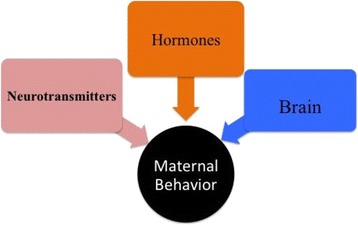
Progression of maternal behaviour

**Fig. 3:**
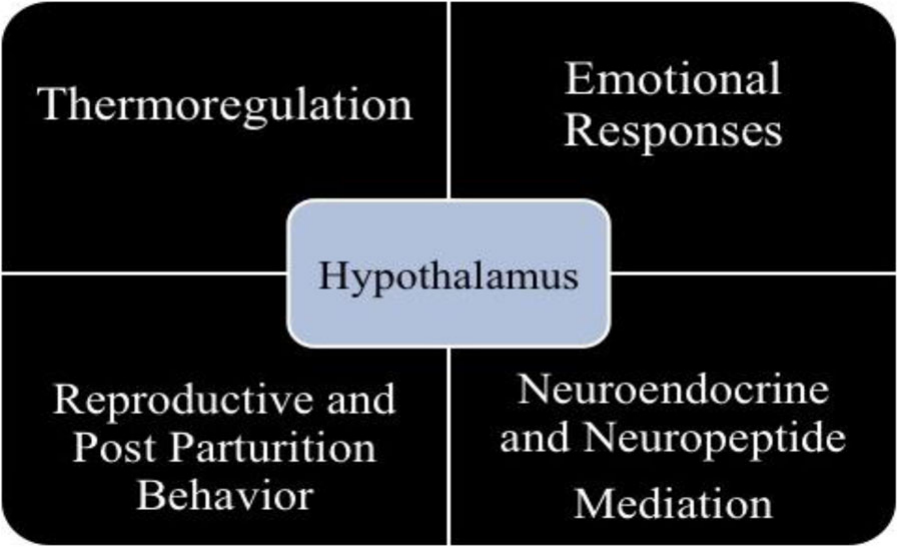
Role of hypothalamus in different post parturition process

Defense of body temperature during exposure to cold requires heat production (shivering and nonshivering thermogenesis) and conservation (peripheral vasoconstriction). Each of these mechanisms are regulated and maintained at different levels of the central nervous system and are influenced by neurotransmitters and hormones (Fig. 2 and Fig. 3). During the present study it has been observed that alteration in ambient temperature affects the thermoregulation which consequently affect the level of stress, anxiety and maternal behaviour [20].

**Fig. 4:**
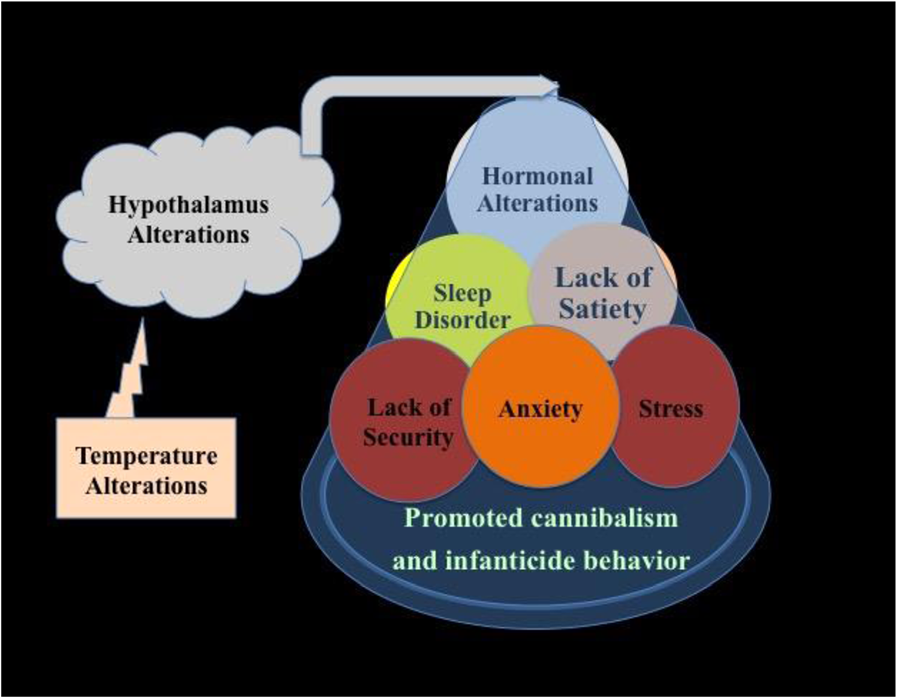
Temperature mediated hypothalamic alteration induced infanticide behaviour

Alteration in ambient temperature was found to affect the temperature recognition center (hypothalamus) in the brain of dams, which contributes in the elevation of postpartum stress and affects the maternal behaviour of dams towards the newborn pups [21]. Alterations homeostasis of hypothalamus affects the coordination capacity of hormones and neurotransmitters [22]. This process results in an elevation of stress, anxiety and lack of satiety along with decreased sense of security in dams postparturition (Fig. 4).

Mice pups are fully dependent on their mother and insulating properties of the nest for nutrition and maintenance of body temperature; to be born in a protected environment is thus crucial for survival. Dead pups also consitutes energy. To eat dead offspring could be considered adaptive; In order to eliminate long lasting unhygenic nest conditions dams sometime consumes their dead pups [13].

Any alteration in nest temperature followed by alteration of body temperature during the early hours of parturion results in discomfort and insecurity, which actively contribute in the infanticide by dam itself [23]. Intrinsic behavioural changes in dams brain followed by hormonal and biochemical imbalance could be a precipitating factor in inciting the cannibalistic habit, which results in infanticide [24]. Present report of mice infanticide by cold stress indicate towards two possibilities. Either pups were died of improper thermoregulation and later on consumed by the dams or dams were failed to maintain their maternal behaviour due to altered temperature conditions and infanticide their living young one’s due to stress. In both the possibilities cold stress remain the culprit to induce infanticide. To critically specify the sequence of events further studies are recommended by authors.

## Conclusions

Based on the results obtained from this study, it is advisable to monitor temperature alterations consistently during delivery of pups and early postpartum phase of mice. Maintenance of temperature within the surroundings plays a crucial role in management of infanticide. Authors strongly recommend the maintenance of body and surrounding temperature within standard limits to prevent infanticidal behaviour and cannibalism within Swiss albino mice population.
